# Cryo-EM structure of a thermophilic encapsulin offers clues to its functions

**DOI:** 10.1107/S2052252521004206

**Published:** 2021-04-24

**Authors:** José R. Castón

**Affiliations:** a Department of Structure of Macromolecules, Centro Nacional de Biotecnología (CNB-CSIC), Campus de Cantoblanco, Madrid, Spain

**Keywords:** cryo-electron microscopy, nanocompartments, encapsulins, thermostability

## Abstract

Wiryaman & Toor [*IUCrJ* (2021). **8**, 342–350] report the cryo-EM structure of a *Thermotoga maritima* encapsulin, a nanocompartment that encapsulates a ferritin-like protein cargo. The 2 Å resolution structure offers insights into the active role of this thermostable encapsulin in regulating iron homeostasis to reduce oxidative stress.

Compartmentalization is an essential strategy, widespread throughout all domains of life Archaea, Bacteria and Eukarya. Although the frontiers between different cell types are becoming more and more diffuse, subcellular structures in eukaryotes rely mainly on membrane-bound organelles, whereas protein-based microcompartments are more prevalent in archaeal and prokaryotic cells. Bacterial protein compartments such as ferritins and carb­oxy­somes have critical roles in the vital processes of iron homeostasis and carbon fixation, respectively. Encapsulins make up another protein nanocompartment that contains one or several cargo enzymes (Jones & Giessen, 2021 ▸); they are broadly extended among prokaryotic and archaeal cells that grow in nearly all terrestrial and aqua­tic habitats including extreme niches, and include bacterial pathogens.

Despite the recent sharp increase in structural studies of encapsulin (Lončar *et al.*, 2020 ▸; Nichols *et al.*, 2021 ▸; Giessen *et al.*, 2019 ▸), these analyses are still in their infancy, and are limited to only two additional systems (Akita *et al.*, 2007 ▸; McHugh *et al.*, 2014 ▸). A seminal study in 2008 from the Ban and Weber–Ban group at the ETH Zurich (Switzerland) reported the 3.1 Å resolution encapsulin structure of *Thermotoga maritima* using X-ray crystallography; they established the structural basis of enzyme encapsulation and, by extension, the true nature of encapsulins (Sutter *et al.*, 2008 ▸). The cargo enzymes are directed to the encapsulin interior through interaction between a targeting peptide of the enzyme and an internal pocket in the encapsulin. Depending on the encapsulated enzymatic core, encapsulin nanocompartment functions are diverse, varying from iron storage to peroxidase-catalyzed oxidative stress reactions.

A distinctive feature of encapsulins is their structural and assembly similarities to viral capsid proteins, although the packaged cargo consists of specific enzymatic activities, rather than nucleic acids. Encapsulins self-assemble into shell-like structures of different sizes (22–42 nm in diameter) with icosahedral symmetry, with triangulation numbers between 1 and 4, although assembly control does not require auxiliary proteins such as scaffolding proteins or proteases. Although they have little sequence similarity, structural comparison between the encapsulin fold and the capsid protein fold of the Hong Kong 97 (HK97)-like viruses (which have the most successful self-replicating system on Earth) shows high structural similarity (Duda & Teschke, 2019 ▸; Suhanovsky & Teschke, 2015 ▸); this indicates that the two compartments share a common evolutionary origin.

In this issue of **IUCrJ**, Wiryaman and Toor at the University of California San Diego report the 2.0 Å resolution encapsulin structure of *T. maritima* (EncTm) using cryo-electron microscopy (cryo-EM) (Wiryaman & Toor, 2021 ▸). Their analysis identifies new structural features of EncTm related to major biological functions of encapsulins. The authors describe the structural basis of the high EncTm thermostability compared with mesophilic encapsulins, as well as a gating mechanism for iron ions in the very porous EncTm nanocompartment, and an unexpected flavin ligand on the outer surface of the protein shell that indicates an active role for EncTm in iron metabolism.

The higher resolution of this structure is observed in the densities of side chains with aromatic rings, in which their associated holes were clearly visible. This is due to the robustness of this assembly, and because the images were acquired in super-resolution mode, which enabled the reconstruction to surpass the physical Nyquist limit (2.0 Å resolution rather than 2.158 Å). Like the fold of the HK97 virus capsid protein, EncTm has three domains: the axial (A) domain, the peripheral (P) domain and the elongated (E) loop. The A domain is responsible for interface interactions at the fivefold symmetry axis, whereas the P domain is at the periphery of pentamers. The E loop is a long β-sheet hairpin with an extended conformation that establishes tight contacts between the twofold symmetry-related subunits from adjacent pentamers; that is, it acts as a molecular hook. An E-loop β-strand forms a β-sheet with a β-strand of the P domain from the other subunit; mostly hydro­phobic and aromatic interactions take place at this interface as well as an ionic interaction, all of which contribute to EncTm thermostability.

The EncTm nanocontainer encapsulates a ferritin-like protein with ferroxidase activity (that is, it stores iron), and has numerous pores on the five- and threefold axes and around the twofold axis, which regulate iron transport through the protein shell. The pores at the fivefold axis, with an ~5 Å diameter hole, have two rings of His and Tyr residues that point towards the exterior and the interior, respectively; although they allow Fe^2+^ diffusion, iron permeability is probably slowed. The pores at the threefold axis, with an ~6 Å diameter hole, have a ring of Phe residues on the interior side that probably blocks iron transport. Finally, pores in the dimer interface only contain hydro­philic residues, which would allow iron transport.

This study further reports the three tricyclic rings, which correspond to flavin ligands, that surround each of the 20 icosahedral threefold axes on the exterior. The flavin binding site is formed by three different subunits, distinct from the threefold-related subunits (Fig. 1 ▸). This unanticipated result indicates that the EncTm shell, in addition to its iron storage function, might have an active role in regulating intracellular iron homeostasis to reduce oxidative stress. Iron is necessary for many essential metabolic routes, but ferrous ions are very reactive and produce toxic hydroxyl radicals; this is sidestepped through their oxidization and retention in the EncTm interior as ferric ions. It is thus no surprise that >6000 encapsulin systems have been identified to date in the cytoplasm of 31 bacterial and four archaeal phyla (Andreas & Giessen, 2021 ▸). These newly identified structural features in EncTm establish the basis for further studies to verify the hypothesis that EncTm-associated flavins participate in iron metabolism. Clarification of this idea will help us to understand encapsulin functions and mechanisms at the molecular level, and will be valuable for new applications in nanotechnology, nanomedicine and materials science.

## Figures and Tables

**Figure 1:**
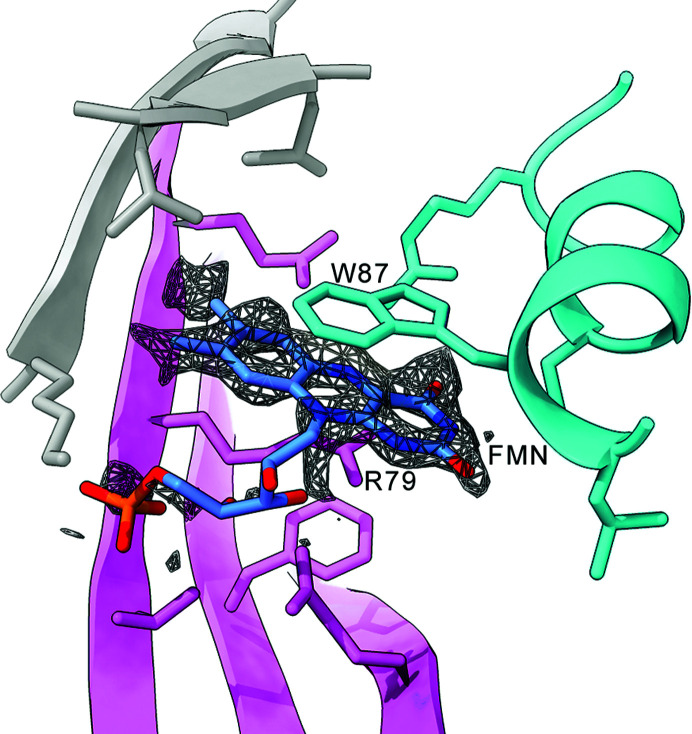
The cryo-EM map of the outer surface of *T. maritima* encapsulin shows a density for a tricyclic ligand (grey mesh), compatible with the modelled flavin mononucleotide (FMN). The binding pocket is formed by three subunits (grey, cyan and pink), and FMN is located between the Trp87 and Arg79 side chains.
